# Double-balloon enteroscopy-guided sclerotherapy for a rare case of blue rubber bleb nevus syndrome

**DOI:** 10.1055/a-2802-4934

**Published:** 2026-04-02

**Authors:** Wentong Lan, Mengfei Xian, Meimei Hu, Yinhui Li, Man Liu, Ning Zhang, Sinan Lin

**Affiliations:** 1 71068 Department of Gastroenterology and Hepatology, The First Affiliated Hospital of Sun Yat-sen University, Guangzhou, China; 2 71068 Department of Medical Ultrasonics, Institute of Diagnostic and Interventional Ultrasound, The First Affiliated Hospital of Sun Yat-sen University, Guangzhou, China

A 16-year-old male patient was admitted due to recurrent melena and fatigue for over 4 years. A gastroscope and a colonoscope at a Vietnamese hospital revealed multiple vascular malformations. Symptoms improved with blooding transfusion and acid suppression, but recurred monthly. Genetic testing identified TEK gene mutation, suggesting a diagnosis of blue rubber bleb nevus syndrome (BRBNS 1 ). On the admission to our center, physical examination demonstrated bluish-purple, rubbery-textured raised lesions on his upper abdomen and medial aspect of the left elbow with pallor skin ( Fig. 1 ). Capsule endoscopy identified bluish raised lesions in multiple segments of the small bowel with active bleeding ( Fig. 2 ). Complete panenteric evaluation was achieved through double-balloon assisted enteroscopy via both antegrade and retrograde approaches, identifying over 10 bluish submucosal protrusions ( Video 1 and Fig. 3 ). Lesions varied in size and morphology, with some exhibiting adenoma-like surface patterns and the largest lesion measured up to 2 cm in diameter. Some lesions showed erosion and recent stigmata of bleeding. To stop the bleeding, endoscopic sclerotherapy was performed with 1–2 ml of polidocanol injected per lesion ( Video 1 and Fig. 4 ). Post-procedurally, melena resolved and hemoglobin increased from 76 g/L to 86 g/L. The patient was then prescribed with sirolimus 2 , as first-line medical therapy for BRBNS, and discharged.

**Fig. 1:**
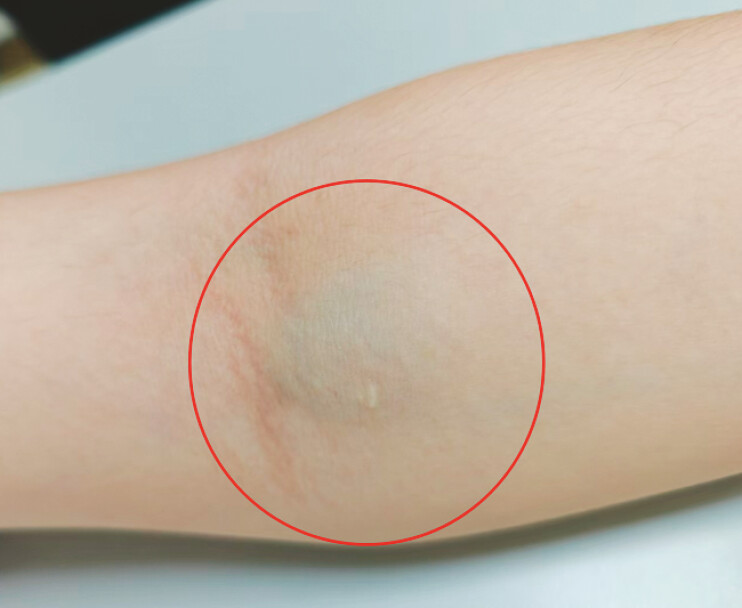
A bluish-purple, rubbery-textured raised lesions on the left elbow.

**Fig. 2:**
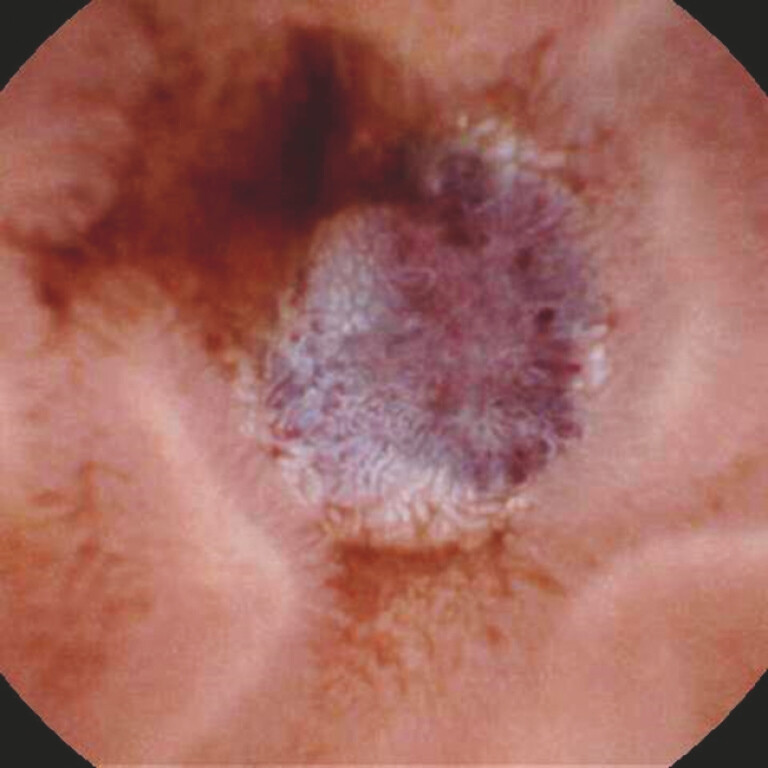
A bluish raised lesion in the small bowel under capsule endoscopy.

More than 10 blue submucosal protrusions (0.8–2 cm) with erythema, erosion, and signs of recent hemorrhage were observed from the duodenum to the ileum. Sclerotherapy (1–2 ml per lesion) was successfully performed, resulting in fading of the lesion color.Video 1

**Fig. 3:**
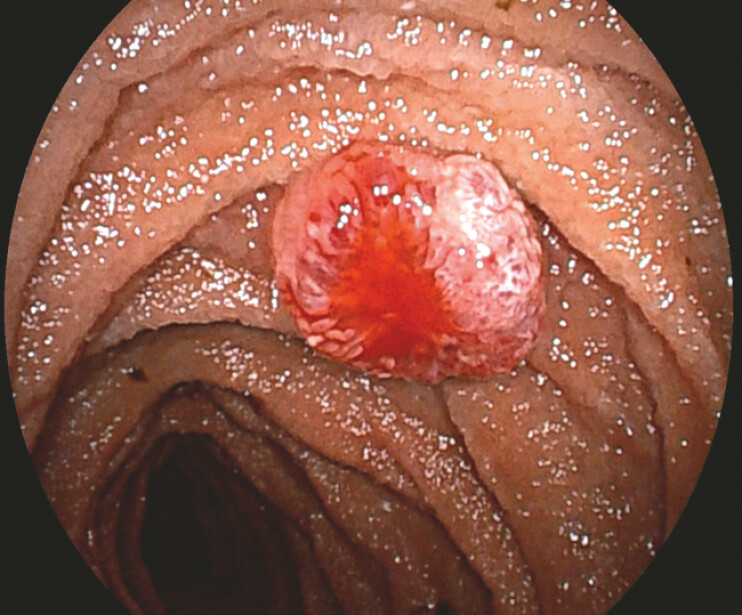
A bluish submucosal lesions with active bleeding observed with double-balloon assisted enteroscopy.

**Fig. 4:**
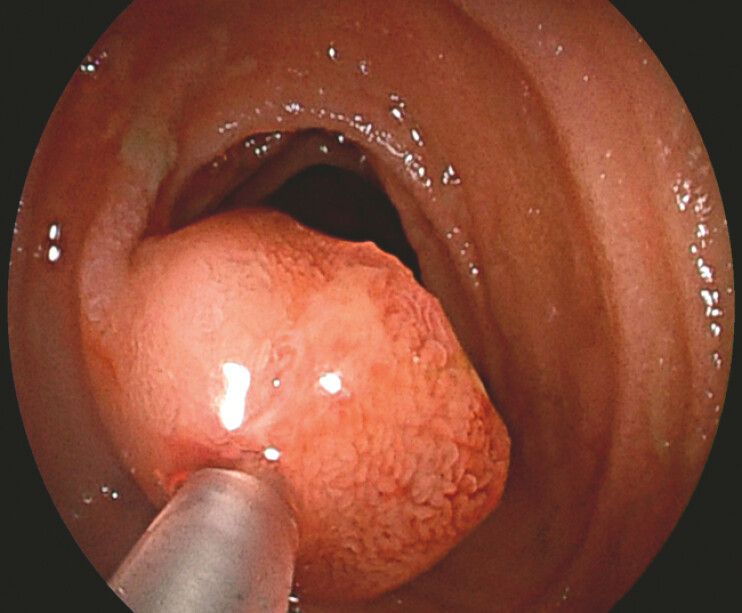
Endoscopic sclerotherapy with polidocanol injection.

Endoscopy_UCTN_Code_TTT_1AP_2AD

## References

[1] Laube R Rickard M Lee AU Gastrointestinal: Small intestinal blue rubber bleb nevus syndrome J Gastroenterol Hepatol 2021 36 2637 10.1111/jgh.15422 33601477

[2] Rimondi A Sorge A Murino A Treatment options for gastrointestinal bleeding blue rubber bleb nevus syndrome: Systematic review Dig Endosc 2024 36 162 171 10.1111/den.14564 37029779 PMC12136259

